# [(1,2,5,6-η)-Cyclo­octa-1,5-diene](4-isopropyl-1-methyl-1,2,4-triazol-5-yl­idene)(tri­cyclo­hexyl­phosphane-κ*P*)iridium(I) tetra­fluorido­borate di­chloro­methane monosolvate

**DOI:** 10.1107/S241431462200685X

**Published:** 2022-07-08

**Authors:** Joshua Rushlow, Andrei V. Astashkin, Daniel R. Albert, Edward Rajaseelan

**Affiliations:** a Department of Chemistry, Millersville University, Millersville, PA 17551, USA; b Department of Chemistry and Biochemistry, The University of Arizona, Tuscon, AZ, 85716, USA; Vienna University of Technology, Austria

**Keywords:** crystal structure, iridium, N-heterocyclic carbenes

## Abstract

The title compound, [Ir(C_8_H_12_)(C_6_H_11_N_3_)(C_18_H_33_P)]BF_4_·CH_2_Cl_2_, a triazole-based N-heterocyclic carbene iridium(I) cationic complex with a tetra­fluorido­borate counter-anion, crystallizes with one di­chloro­methane solvent mol­ecule per formula unit. The Ir^I^ atom of the cationic complex has a distorted square-planar coordination environment, defined by a bidentate cyclo­octa-1,5-diene (COD) ligand, an N-heterocyclic carbene, and a tri­cyclo­hexyl­phosphane ligand. The complex crystallizes in a *C*-centered monoclinic unit cell and has an unusually high number of eight formula units.

## Abstract 2

The IrI atom in the complex cation of the title salt has a distorted square-planar coordination environment, defined by a bidentate cyclo­octa-1,5-diene (COD) ligand, an N-heterocyclic carbene, and a tri­cyclo­hexyl­phosphane ligand.

## Graphical abstract

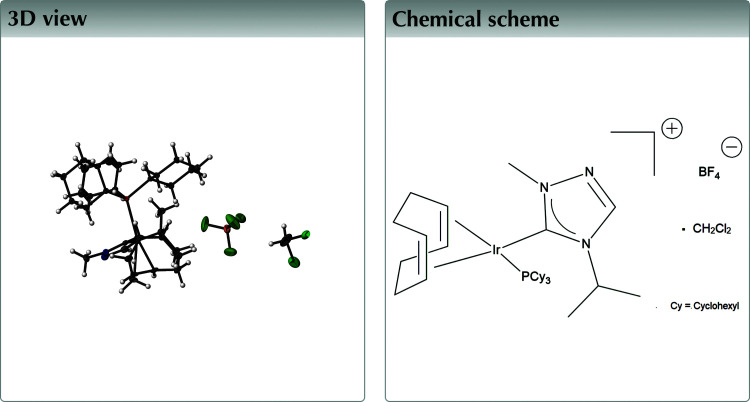

## Structure description

N-heterocyclic carbenes (NHCs) have emerged as excellent spectator ligands in homogeneous catalysis (Cazin, 2013 ▸; de Frémont *et al.*, 2009 ▸; Díez-Gonzáles *et al.*, 2009 ▸; Rovis & Nolan, 2013 ▸; Ruff *et al.*, 2016 ▸; Zuo *et al.*, 2014 ▸). Their catalytic activities in the transfer hydrogenation of ketones and imines have also been studied and reported (Albrecht *et al.*, 2002 ▸; Gnanamgari *et al.*, 2007 ▸). The NHC ligands can be tuned sterically and electronically by having different substituents on the nitro­gen atoms (Gusev, 2009 ▸). Many imidazole- and triazole-based NHC rhodium and iridium complexes have been synthesized and structurally characterized (Herrmann *et al.*, 2006 ▸; Wang & Lin, 1998 ▸; Chianese *et al.*, 2004 ▸). As part of our ongoing research, we continue to synthesize new imidazole- and triazole-based NHC complexes of rhodium and iridium to study the effect of different substituents on the NHCs and co-ligands coordinating to the transition metal in transfer hydrogenation reactions (Nichol *et al.*, 2009 ▸, 2010 ▸, 2011 ▸, 2012 ▸; Idrees *et al.*, 2017*a* ▸,*b* ▸; Rood *et al.*, 2021 ▸; Rushlow *et al.*, 2021 ▸; Newman *et al.*, 2021 ▸; Castaldi *et al.*, 2021 ▸).

**Figure 1:**
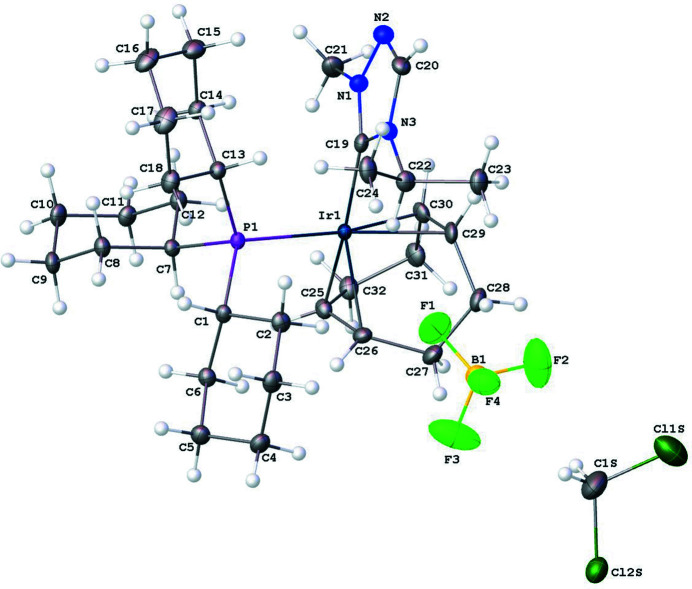
The mol­ecular entities in the crystal structure of the title compound (**4**). Displacement ellipsoids are drawn at the 50% probability level.

**Figure 2:**
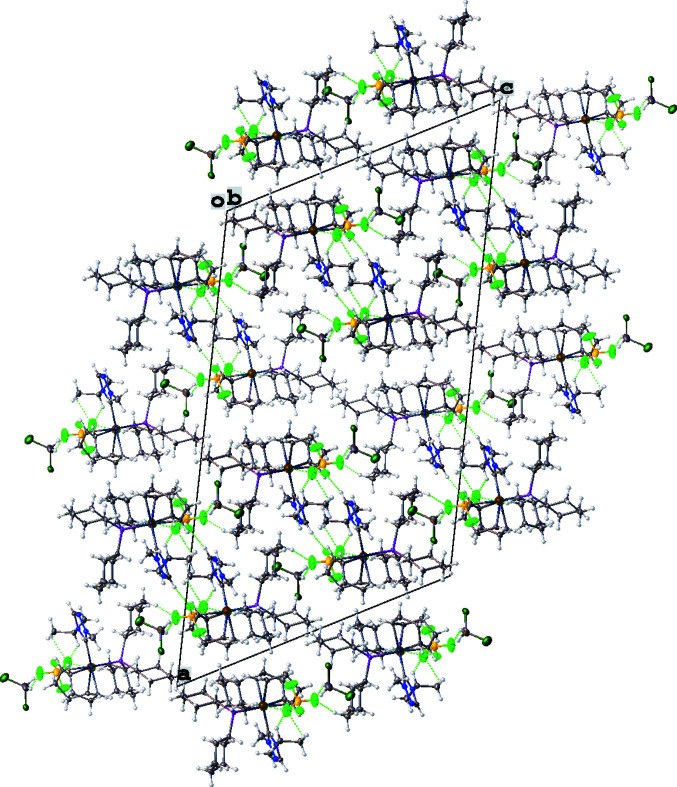
Crystal packing unit-cell diagram of the title compound (**4**) shown along the *b* axis. Non-classical hydrogen-bonding inter­actions between F and H atoms are shown as dotted green lines.

The mol­ecular structure of the title complex, [Ir(C_8_H_12_)(C_18_H_33_P)(C_6_H_11_N_3_)][BF_4_]·CH_2_Cl_2_ (**4**), comprises an Ir^I^ cationic complex, a tetra­fluorido­borate counter-anion, and a CH_2_Cl_2_ solvent mol­ecule, as illustrated in Fig. 1 ▸. The coordination sphere around the Ir^I^ atom, formed by the bidentate (1,2,5,6-η)-cyclo­octa-1,5-diene (COD), NHC, and tri­cyclo­hexyl­phosphane ligands, results in a distorted square-planar shape. The N1—C19_(NHC)_—N3 bond angle in the triazole-based carbene is 102.8 (2)° deviating from the expected 120° for the *sp* ^2^-hybridized carbon atom. Other selected bond lengths and angles in the structure are: Ir1—C19_(NHC)_ = 2.035 (3) Å, Ir1—P1 = 2.3732 (7) Å, and C19—Ir1—P1 = 94.07 (7)°. The cyclo­hexyl rings of the tri­cyclo­hexyl­phosphane ligand are all in the expected chair conformation, with the mean planes of the cyclo­hexyl rings forming dihedral angles of 59.70 (15), 61.77 (14), and 83.20 (15)°. The crystal packing diagram of the title compound is shown in Fig. 2 ▸. Several close C—H⋯F contacts stabilizing the orientation of the [BF_4_]^−^ group with the iridium(I) complex and di­chloro­methane solvate are reported in Table 1 ▸. These non-classical hydrogen-bonds are shown as green dotted lines in Fig. 2 ▸.

## Synthesis and crystallization

**Figure 3:**
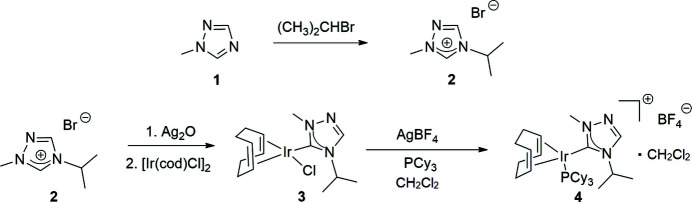
Reaction scheme for the synthesis of the N-heterocyclic carbene (**2**) and subsequent formation of the title compound (**4**).

**1-Methyl-1,2,4 triazole** (**1**) was purchased from Matrix Scientific. All other compounds used in the syntheses as shown in Fig. 3 ▸ were obtained from Sigma-Aldrich and Strem and used as received. All subsequent synthesis procedures were performed under nitro­gen using reagent grade solvents, which were used as received without further purification. NMR spectra were recorded at room temperature in CDCl_3_ on a 400 MHz Varian spectrometer (operating at 162 MHz for ^31^P) and referenced to the residual solvent peak (δ in ppm).

**4-Isopropyl-1-methyl-1,2,4-triazolium bromide (2)**: 1-meth­yl-1,2,4-triazole (**1**) (5.01 g, 60.28 mmol) and isopropyl bromide (10.48 g, 85.2 mmol) were added to toluene (20 ml), and the mixture was refluxed for 48 h. Once cooled, the liquid was deca­nted, the white solid product that had formed was washed with ether, filtered, and dried. Yield: 2.48 g (20%). ^1^H NMR: δ 11.79 (*s*, 1 H, N—C_5_H—N), 8.97 (*s*, 1 H, N—C_3_H—N), 5.83 [*m*, 1 H, CH(CH_3_)_2_], 4.29 (*s*, 3 H, N—CH_3_), 1.73 [*d*, 6 H, CH(CH_3_)_2_]. ^13^C NMR: δ 143.52 (N—CH—N), 142.65 (N—CH—N), 53.32 [CH(CH_3_)_2_], 39.62 (N—CH_3_), 23.21[CH(CH_3_)_2_].

**[(1,2,5,6-η)-Cyclo­octa-1,5-diene](4-isopropyl-1-methyl-1,2,4-triazol-5-yl­idene) chloro­iridium (3)**: 4-ispopropyl-1-methyl-1,2,4 triazolium bromide (**2**) (0.061g, 0.300 mmol), Ag_2_O (0.035 g, 0.149 mmol), and 10 ml of CH_2_Cl_2_ were added to an oven-dried flask and stirred under N_2_ in the dark for 90 min. The mixture was filtered through Celite into [Ir(COD)Cl]_2_ (0.100 g, 0.149 mmol) and stirred in the dark for 90 min. The resulting mixture was filtered through Celite and the solvent was removed under reduced pressure. The red solid product was washed with pentane and allowed to dry overnight under vacuum. Yield: 0.139 g (100%). ^1^H NMR: δ 7.91 (*s*, 1 H, N—C_3_H—N), 5.52 [*m*, 1 H, CH(CH_3_)_2_], 4.74, 4.76 (*m*, 4 H, CH of COD) 4.09 (*s*, 3 H, CH_3_—N), 2.96, 2.30, 2.17, 1.86 (*m*, 8 H, CH_2_ of COD),1.28 [*m*, 6 H, CH(CH_3_)_2_]. ^13^C NMR: δ 181.21 (Ir—C), 138.79 (N—CH—N), 86.59, 85.96 (CH of COD), 51.73 [CH(CH_3_)_2_], 40.52 (N—CH_3_), 33.72, 33.28, 29.65, 29.24 (CH_2_ of COD), 24.17, 23.31[CH(CH_3_)_2_].

**[(1,2,5,6-η)-Cyclo­octa-1,5-diene](4-isopropyl-1-methyl-1,2,4-triazol-5-yl­idene)(tri­cyclo­hexyl­phosphane)iridium(I) tetra­fluorido­borate (4)**: Tri­cyclo­hexyl­phosphane (0.085 g, 0.308 mmol) and AgBF_4_ (0.059 g 0.308 mmol) were added to an oven-dried flask containing complex **3** (0.140 g, 0.308 mmol) in 10 ml of CH_2_Cl_2_, and stirred under N_2_ in the dark for 90 mins. The mixture was filtered through Celite and the solvent was removed under reduced pressure. The bright-red solid was washed with pentane and dried under vacuum. Yield: 0.214 g (100%). ^1^H NMR: δ 8.58 (*s*, 1 H, N—C_3_H—N), 5.29 [*m*, 1 H, CH(CH_3_)_2_], 4.38, 3.99 (*m*, 4 H, CH of COD), 4.05 (*s*, 3 H, CH_3_—N), 2.27–0.86 CH_2_ of COD and cyclo­hexyl, 1.21 [*m*, 6 H, CH(CH_3_)_2_]. ^13^C NMR: δ 178.26 (Ir—C), 142.01 (N—CH—N), 77.30, 77.19, 76.98, 77.66 (CH of COD), 54.23 [CH(CH_3_)_2_], 40.51 (N—CH_3_), 34.94, 34.10, 30.36, 30.14 (CH_2_ of COD), 27.79–25.92 (CH_2_ of cyclo­hex­yl), 24.50, 22.86 [CH(CH_3_)_2_]. ^31^P: δ 39.81.

The title compound (**4**) was crystallized by slow diffusion of pentane into a CH_2_Cl_2_ solution.

## Refinement

Crystal data, data collection, and structure refinement details are summarized in Table 2 ▸.

## Supplementary Material

Crystal structure: contains datablock(s) I. DOI: 10.1107/S241431462200685X/wm4168sup1.cif

Structure factors: contains datablock(s) I. DOI: 10.1107/S241431462200685X/wm4168Isup2.hkl

CCDC reference: 2184164

Additional supporting information:  crystallographic information; 3D view; checkCIF report

## Figures and Tables

**Table 1:** Hydrogen-bond geometry (Å, °)

*D*—H⋯*A*	*D*—H	H⋯*A*	*D*⋯*A*	*D*—H⋯*A*
C2—H2*A*⋯F1	0.99	2.54	3.204 (4)	124
C23—H23*C*⋯F1	0.98	2.51	3.137 (4)	122
C20—H20⋯F4^i^	0.95	2.49	3.400 (3)	161
C30—H30⋯F4^ii^	1.00	2.35	3.192 (3)	141
C16—H16*B*⋯F2^iii^	0.99	2.54	3.404 (4)	146
C22—H22⋯F1	1.00	2.41	3.094 (4)	125
C31—H31*B*⋯F3^ii^	0.99	2.35	3.330 (4)	170
C1*S*—H1*SA*⋯F2	0.99	2.50	3.362 (5)	145

Symmetry codes: (i) 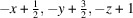 ; (ii) 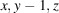 ; (iii) 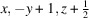 .

**Table 2:** Experimental details

Crystal data	
Chemical formula	[Ir(C_8_H_12_)(C_6_H_11_N_3_)(C_18_H_33_P)]BF_4_·CH_2_Cl_2_
*M*_r_	877.70
Crystal system, space group	Monoclinic, *C*2/*c*
Temperature (K)	100
*a*, *b*, *c* (Å)	35.832 (5), 10.2608 (15), 22.034 (3)
β (°)	118.095 (3)
*V* (Å^3^)	7146.7 (17)
*Z*	8
Radiation type	Mo *K*α
μ (mm^−1^)	3.98
Crystal size (mm)	0.27 × 0.11 × 0.07

Data collection	
Diffractometer	Bruker APEXII CCD
Absorption correction	Multi-scan (*SADABS*; Krause *et al.*, 2015 ▸)
*T*_min_, *T*_max_	0.555, 0.746
No. of measured, independent and observed [*I* > 2σ(*I*)] reflections	151141, 7958, 7086
*R*_int_	0.061
(sin θ/λ)_max_ (Å^−1^)	0.643

Refinement	
*R*[*F* ^2^ > 2σ(*F* ^2^)], *wR*(*F* ^2^), *S*	0.022, 0.052, 1.05
No. of reflections	7958
No. of parameters	409
No. of restraints	1
H-atom treatment	H-atom parameters constrained
Δρ_max_, Δρ_min_ (e Å^−3^)	1.83, −0.69

Computer programs: *APEX2* and *SAINT* (Bruker, 2007 ▸), *olex2.solve* (Bourhis *et al.*, 2015 ▸), *SHELXL* (Sheldrick, 2015 ▸), *OLEX2* (Dolomanov *et al.*, 2009 ▸), and *publCIF* (Westrip, 2010 ▸).

## full crystallographic data

### Crystal data

**Table:**

[Ir(C_8_H_12_)(C_6_H_11_N_3_)(C_18_H_33_P)]BF_4_·CH_2_Cl_2_	*F*(000) = 3552
*M**_r_* = 877.70	*D*_x_ = 1.631 Mg m^−^^3^
Monoclinic, *C*2/*c*	Mo *K*α radiation, λ = 0.71073 Å
*a* = 35.832 (5) Å	Cell parameters from 9640 reflections
*b* = 10.2608 (15) Å	θ = 2.5–27.1°
*c* = 22.034 (3) Å	µ = 3.98 mm^−^^1^
β = 118.095 (3)°	*T* = 100 K
*V* = 7146.7 (17) Å^3^	Plate, clear light pink
*Z* = 8	0.27 × 0.11 × 0.07 mm

### Data collection

**Table:**

Bruker APEXII CCD diffractometer	7086 reflections with *I* > 2σ(*I*)
φ and ω scans	*R*_int_ = 0.061
Absorption correction: multi-scan (SADABS; Krause *et al.*, 2015)	θ_max_ = 27.2°, θ_min_ = 1.3°
*T*_min_ = 0.555, *T*_max_ = 0.746	*h* = −45→46
151141 measured reflections	*k* = −13→13
7958 independent reflections	*l* = −28→27

### Refinement

**Table:**

Refinement on *F*^2^	Primary atom site location: iterative
Least-squares matrix: full	Hydrogen site location: inferred from neighbouring sites
*R*[*F*^2^ > 2σ(*F*^2^)] = 0.022	H-atom parameters constrained
*wR*(*F*^2^) = 0.052	*w* = 1/[σ^2^(*F*_o_^2^) + (0.0234*P*)^2^ + 23.3944*P*] where *P* = (*F*_o_^2^ + 2*F*_c_^2^)/3
*S* = 1.05	(Δ/σ)_max_ = 0.004
7958 reflections	Δρ_max_ = 1.83 e Å^−^^3^
409 parameters	Δρ_min_ = −0.69 e Å^−^^3^
1 restraint	

### Special details

**Table:**

Geometry. All esds (except the esd in the dihedral angle between two l.s. planes) are estimated using the full covariance matrix. The cell esds are taken into account individually in the estimation of esds in distances, angles and torsion angles; correlations between esds in cell parameters are only used when they are defined by crystal symmetry. An approximate (isotropic) treatment of cell esds is used for estimating esds involving l.s. planes.

### Fractional atomic coordinates and isotropic or equivalent isotropic displacement parameters (Å^2^)

**Table:**

	*x*	*y*	*z*	*U*_iso_*/*U*_eq_	
Ir1	0.37947 (2)	0.36533 (2)	0.66268 (2)	0.01104 (4)	
P1	0.39296 (2)	0.44263 (6)	0.77266 (3)	0.01133 (12)	
Cl2S	0.40895 (2)	1.15711 (8)	0.42611 (4)	0.02888 (16)	
Cl1S	0.32454 (3)	1.06150 (10)	0.33196 (5)	0.0487 (2)	
F4	0.33674 (6)	0.95299 (19)	0.51922 (11)	0.0381 (5)	
N1	0.29702 (7)	0.2187 (2)	0.62775 (11)	0.0165 (5)	
N3	0.28433 (7)	0.4161 (2)	0.59694 (11)	0.0148 (4)	
F2	0.34763 (10)	0.7797 (2)	0.46717 (12)	0.0665 (8)	
F3	0.40161 (8)	0.8615 (2)	0.56303 (16)	0.0674 (8)	
C19	0.31703 (8)	0.3312 (2)	0.63092 (13)	0.0140 (5)	
N2	0.25339 (7)	0.2280 (2)	0.59297 (12)	0.0206 (5)	
F1	0.34932 (10)	0.7542 (3)	0.56918 (13)	0.0762 (9)	
C1	0.41482 (8)	0.6111 (2)	0.79339 (13)	0.0133 (5)	
H1	0.409073	0.642915	0.831081	0.016*	
C7	0.43280 (8)	0.3465 (2)	0.84639 (13)	0.0124 (5)	
H7	0.460500	0.366415	0.847728	0.015*	
C13	0.34343 (8)	0.4505 (2)	0.78081 (13)	0.0141 (5)	
H13	0.320559	0.472551	0.733675	0.017*	
C6	0.46335 (8)	0.6214 (2)	0.82126 (11)	0.0148 (5)	
H6A	0.478006	0.573466	0.865319	0.018*	
H6B	0.471149	0.580549	0.788106	0.018*	
C12	0.42781 (8)	0.1971 (2)	0.83749 (13)	0.0150 (5)	
H12A	0.401002	0.169971	0.836486	0.018*	
H12B	0.426543	0.171706	0.793210	0.018*	
C2	0.38961 (8)	0.7009 (2)	0.73112 (14)	0.0163 (5)	
H2A	0.391499	0.666680	0.690660	0.020*	
H2B	0.359446	0.700389	0.720122	0.020*	
C21	0.31663 (9)	0.0937 (3)	0.65715 (15)	0.0221 (6)	
H21A	0.310806	0.031417	0.620001	0.033*	
H21B	0.304984	0.060358	0.686421	0.033*	
H21C	0.347268	0.105237	0.684765	0.033*	
C25	0.44696 (8)	0.3374 (3)	0.69905 (14)	0.0167 (5)	
H25	0.465054	0.353427	0.749386	0.020*	
C8	0.43839 (8)	0.3886 (3)	0.91742 (13)	0.0158 (5)	
H8A	0.443145	0.483866	0.922958	0.019*	
H8B	0.412273	0.368539	0.920393	0.019*	
C23	0.26856 (9)	0.5766 (3)	0.50488 (14)	0.0224 (6)	
H23A	0.237706	0.572301	0.485058	0.034*	
H23B	0.278255	0.509140	0.484080	0.034*	
H23C	0.276744	0.662600	0.495709	0.034*	
C11	0.46487 (9)	0.1279 (2)	0.89647 (13)	0.0158 (5)	
H11A	0.460147	0.032577	0.891400	0.019*	
H11B	0.491179	0.147151	0.893945	0.019*	
C9	0.47579 (9)	0.3179 (3)	0.97497 (13)	0.0172 (5)	
H9A	0.478191	0.343834	1.019954	0.021*	
H9B	0.502223	0.343751	0.974335	0.021*	
C18	0.34015 (9)	0.5541 (3)	0.82801 (14)	0.0193 (6)	
H18A	0.346227	0.640997	0.815137	0.023*	
H18B	0.361480	0.536406	0.876193	0.023*	
C20	0.24711 (8)	0.3498 (3)	0.57501 (14)	0.0185 (6)	
H20	0.219940	0.388176	0.549698	0.022*	
C26	0.43439 (8)	0.4508 (3)	0.65866 (11)	0.0168 (5)	
H26	0.444608	0.531772	0.687085	0.020*	
C32	0.45361 (9)	0.2064 (3)	0.67450 (14)	0.0192 (6)	
H32A	0.452366	0.137592	0.705011	0.023*	
H32B	0.482169	0.203978	0.677949	0.023*	
C4	0.45350 (9)	0.8468 (3)	0.76842 (15)	0.0197 (6)	
H4A	0.463430	0.938160	0.778773	0.024*	
H4B	0.459077	0.815192	0.731018	0.024*	
C14	0.33119 (8)	0.3160 (3)	0.79709 (14)	0.0160 (5)	
H14A	0.332548	0.250194	0.765315	0.019*	
H14B	0.351701	0.290493	0.844618	0.019*	
C30	0.37730 (9)	0.2283 (3)	0.58452 (13)	0.0177 (5)	
H30	0.355490	0.158421	0.572558	0.021*	
C10	0.47030 (9)	0.1703 (3)	0.96657 (14)	0.0179 (5)	
H10A	0.495397	0.126619	1.003385	0.022*	
H10B	0.445176	0.143510	0.971083	0.022*	
C16	0.28327 (10)	0.4211 (3)	0.83670 (16)	0.0258 (6)	
H16A	0.253819	0.424430	0.829333	0.031*	
H16B	0.301900	0.397242	0.885332	0.031*	
C22	0.28870 (9)	0.5548 (3)	0.58210 (14)	0.0174 (5)	
H22	0.319547	0.575082	0.602327	0.021*	
C15	0.28669 (9)	0.3177 (3)	0.78991 (16)	0.0231 (6)	
H15A	0.265871	0.336313	0.741537	0.028*	
H15B	0.280048	0.231093	0.802164	0.028*	
C29	0.36078 (9)	0.3470 (3)	0.55232 (13)	0.0181 (6)	
H29	0.329489	0.346033	0.521651	0.022*	
C5	0.47773 (9)	0.7629 (3)	0.83248 (14)	0.0187 (6)	
H5A	0.508287	0.766504	0.846153	0.022*	
H5B	0.473836	0.799412	0.870709	0.022*	
C31	0.42068 (9)	0.1769 (3)	0.60030 (14)	0.0205 (6)	
H31A	0.429634	0.217383	0.568440	0.025*	
H31B	0.419020	0.081538	0.592699	0.025*	
C3	0.40612 (9)	0.8415 (3)	0.74491 (14)	0.0192 (6)	
H3A	0.390892	0.893617	0.702391	0.023*	
H3B	0.400367	0.880384	0.780789	0.023*	
C24	0.26977 (9)	0.6436 (3)	0.61597 (14)	0.0218 (6)	
H24A	0.275798	0.734726	0.610367	0.033*	
H24B	0.282248	0.622876	0.665071	0.033*	
H24C	0.239084	0.630457	0.594223	0.033*	
C28	0.38413 (9)	0.4454 (3)	0.53192 (14)	0.0221 (6)	
H28A	0.368542	0.529057	0.520982	0.026*	
H28B	0.384614	0.414669	0.489710	0.026*	
C27	0.42968 (9)	0.4688 (3)	0.58804 (14)	0.0218 (6)	
H27A	0.448791	0.407585	0.581380	0.026*	
H27B	0.438268	0.558577	0.583636	0.026*	
C17	0.29586 (10)	0.5546 (3)	0.82245 (16)	0.0252 (6)	
H17A	0.295162	0.618232	0.855678	0.030*	
H17B	0.275024	0.582953	0.775659	0.030*	
B1	0.35915 (13)	0.8357 (4)	0.53007 (19)	0.0317 (8)	
C1S	0.36391 (13)	1.0744 (5)	0.4171 (2)	0.0568 (12)	
H1SA	0.372205	0.985789	0.436656	0.068*	
H1SB	0.352286	1.120661	0.443906	0.068*	

### Atomic displacement parameters (Å^2^)

**Table:**

	*U*^11^	*U*^22^	*U*^33^	*U*^12^	*U*^13^	*U*^23^
Ir1	0.01177 (5)	0.01212 (5)	0.00905 (5)	0.00001 (4)	0.00476 (4)	−0.00061 (4)
P1	0.0132 (3)	0.0105 (3)	0.0100 (3)	0.0009 (2)	0.0053 (3)	0.0001 (2)
Cl2S	0.0298 (4)	0.0360 (4)	0.0242 (4)	0.0020 (3)	0.0155 (3)	0.0033 (3)
Cl1S	0.0400 (5)	0.0429 (5)	0.0466 (5)	−0.0068 (4)	0.0067 (4)	−0.0053 (4)
F4	0.0372 (11)	0.0322 (10)	0.0466 (12)	−0.0052 (9)	0.0210 (10)	−0.0079 (9)
N1	0.0170 (11)	0.0167 (11)	0.0158 (11)	−0.0025 (9)	0.0077 (9)	0.0002 (9)
N3	0.0132 (11)	0.0166 (11)	0.0140 (11)	0.0006 (9)	0.0059 (9)	−0.0001 (9)
F2	0.113 (2)	0.0406 (13)	0.0361 (13)	0.0102 (14)	0.0270 (14)	−0.0078 (10)
F3	0.0350 (13)	0.0366 (13)	0.109 (2)	−0.0045 (10)	0.0166 (14)	−0.0099 (13)
C19	0.0168 (13)	0.0161 (12)	0.0102 (12)	0.0004 (10)	0.0072 (10)	−0.0002 (9)
N2	0.0167 (12)	0.0261 (13)	0.0190 (12)	−0.0035 (10)	0.0083 (10)	−0.0009 (10)
F1	0.091 (2)	0.0757 (19)	0.0468 (15)	−0.0387 (16)	0.0203 (14)	0.0237 (13)
C1	0.0173 (13)	0.0107 (12)	0.0120 (12)	−0.0002 (9)	0.0069 (10)	−0.0010 (9)
C7	0.0117 (12)	0.0128 (12)	0.0112 (12)	0.0013 (9)	0.0040 (10)	0.0013 (9)
C13	0.0140 (12)	0.0164 (12)	0.0126 (12)	0.0004 (10)	0.0068 (10)	−0.0006 (10)
C6	0.0161 (13)	0.0134 (12)	0.0142 (12)	0.0009 (10)	0.0066 (10)	−0.0009 (9)
C12	0.0186 (13)	0.0124 (12)	0.0133 (13)	0.0024 (10)	0.0070 (11)	0.0007 (10)
C2	0.0174 (13)	0.0136 (12)	0.0161 (13)	0.0030 (10)	0.0064 (11)	0.0019 (10)
C21	0.0269 (15)	0.0148 (13)	0.0214 (14)	−0.0034 (11)	0.0086 (12)	0.0014 (11)
C25	0.0150 (13)	0.0224 (14)	0.0135 (13)	0.0005 (10)	0.0073 (11)	−0.0042 (10)
C8	0.0177 (13)	0.0162 (13)	0.0120 (12)	0.0011 (10)	0.0059 (11)	−0.0009 (10)
C23	0.0260 (15)	0.0234 (14)	0.0151 (14)	0.0054 (12)	0.0074 (12)	0.0033 (11)
C11	0.0201 (13)	0.0122 (12)	0.0158 (13)	0.0034 (10)	0.0092 (11)	0.0029 (10)
C9	0.0193 (13)	0.0198 (13)	0.0096 (12)	0.0011 (11)	0.0043 (11)	0.0002 (10)
C18	0.0240 (15)	0.0192 (13)	0.0173 (14)	0.0037 (11)	0.0119 (12)	−0.0006 (10)
C20	0.0116 (12)	0.0263 (15)	0.0156 (13)	−0.0013 (11)	0.0048 (11)	−0.0007 (11)
C26	0.0159 (13)	0.0194 (13)	0.0183 (13)	−0.0043 (10)	0.0107 (11)	−0.0026 (10)
C32	0.0175 (13)	0.0235 (14)	0.0169 (13)	0.0057 (11)	0.0084 (11)	−0.0011 (11)
C4	0.0243 (15)	0.0178 (13)	0.0193 (14)	−0.0028 (11)	0.0120 (12)	0.0014 (11)
C14	0.0172 (13)	0.0171 (12)	0.0151 (13)	0.0002 (10)	0.0086 (11)	0.0004 (10)
C30	0.0187 (14)	0.0202 (13)	0.0131 (13)	−0.0028 (10)	0.0067 (11)	−0.0080 (10)
C10	0.0213 (14)	0.0176 (13)	0.0129 (13)	0.0043 (11)	0.0065 (11)	0.0051 (10)
C16	0.0239 (15)	0.0332 (16)	0.0294 (16)	0.0060 (13)	0.0200 (13)	0.0065 (13)
C22	0.0180 (13)	0.0152 (13)	0.0174 (13)	0.0034 (10)	0.0071 (11)	0.0031 (10)
C15	0.0162 (14)	0.0282 (15)	0.0259 (15)	−0.0001 (11)	0.0108 (12)	0.0041 (12)
C29	0.0189 (13)	0.0272 (15)	0.0085 (12)	−0.0007 (11)	0.0068 (11)	−0.0033 (10)
C5	0.0216 (14)	0.0166 (13)	0.0175 (14)	−0.0031 (11)	0.0089 (12)	−0.0017 (10)
C31	0.0244 (15)	0.0197 (13)	0.0183 (14)	0.0015 (11)	0.0107 (12)	−0.0072 (11)
C3	0.0253 (15)	0.0140 (13)	0.0177 (14)	0.0014 (11)	0.0097 (12)	0.0029 (10)
C24	0.0255 (15)	0.0212 (14)	0.0143 (13)	0.0087 (11)	0.0059 (12)	0.0024 (11)
C28	0.0255 (15)	0.0291 (15)	0.0140 (13)	0.0020 (12)	0.0113 (12)	0.0038 (11)
C27	0.0252 (15)	0.0256 (15)	0.0207 (14)	−0.0042 (12)	0.0160 (13)	0.0000 (11)
C17	0.0277 (16)	0.0267 (15)	0.0283 (16)	0.0113 (12)	0.0190 (14)	0.0037 (12)
B1	0.041 (2)	0.0238 (18)	0.0246 (19)	−0.0130 (15)	0.0110 (17)	0.0005 (14)
C1S	0.048 (2)	0.096 (4)	0.034 (2)	−0.021 (2)	0.0252 (19)	0.004 (2)

### Geometric parameters (Å, º)

**Table:**

Ir1—P1	2.3732 (7)	C11—H11B	0.9900
Ir1—C19	2.035 (3)	C11—C10	1.526 (4)
Ir1—C25	2.179 (3)	C9—H9A	0.9900
Ir1—C26	2.194 (3)	C9—H9B	0.9900
Ir1—C30	2.196 (3)	C9—C10	1.528 (4)
Ir1—C29	2.205 (3)	C18—H18A	0.9900
P1—C1	1.863 (3)	C18—H18B	0.9900
P1—C7	1.862 (3)	C18—C17	1.533 (4)
P1—C13	1.868 (3)	C20—H20	0.9500
Cl2S—C1S	1.750 (4)	C26—H26	1.0000
Cl1S—C1S	1.742 (4)	C26—C27	1.496 (3)
F4—B1	1.403 (5)	C32—H32A	0.9900
N1—C19	1.343 (3)	C32—H32B	0.9900
N1—N2	1.383 (3)	C32—C31	1.530 (4)
N1—C21	1.460 (3)	C4—H4A	0.9900
N3—C19	1.366 (3)	C4—H4B	0.9900
N3—C20	1.367 (3)	C4—C5	1.528 (4)
N3—C22	1.484 (3)	C4—C3	1.525 (4)
F2—B1	1.372 (4)	C14—H14A	0.9900
F3—B1	1.368 (5)	C14—H14B	0.9900
N2—C20	1.298 (4)	C14—C15	1.527 (4)
F1—B1	1.361 (4)	C30—H30	1.0000
C1—H1	1.0000	C30—C29	1.394 (4)
C1—C6	1.551 (4)	C30—C31	1.519 (4)
C1—C2	1.543 (3)	C10—H10A	0.9900
C7—H7	1.0000	C10—H10B	0.9900
C7—C12	1.546 (3)	C16—H16A	0.9900
C7—C8	1.543 (3)	C16—H16B	0.9900
C13—H13	1.0000	C16—C15	1.524 (4)
C13—C18	1.530 (4)	C16—C17	1.520 (4)
C13—C14	1.541 (4)	C22—H22	1.0000
C6—H6A	0.9900	C22—C24	1.525 (4)
C6—H6B	0.9900	C15—H15A	0.9900
C6—C5	1.522 (3)	C15—H15B	0.9900
C12—H12A	0.9900	C29—H29	1.0000
C12—H12B	0.9900	C29—C28	1.509 (4)
C12—C11	1.527 (3)	C5—H5A	0.9900
C2—H2A	0.9900	C5—H5B	0.9900
C2—H2B	0.9900	C31—H31A	0.9900
C2—C3	1.534 (4)	C31—H31B	0.9900
C21—H21A	0.9800	C3—H3A	0.9900
C21—H21B	0.9800	C3—H3B	0.9900
C21—H21C	0.9800	C24—H24A	0.9800
C25—H25	1.0000	C24—H24B	0.9800
C25—C26	1.404 (4)	C24—H24C	0.9800
C25—C32	1.510 (4)	C28—H28A	0.9900
C8—H8A	0.9900	C28—H28B	0.9900
C8—H8B	0.9900	C28—C27	1.533 (4)
C8—C9	1.526 (4)	C27—H27A	0.9900
C23—H23A	0.9800	C27—H27B	0.9900
C23—H23B	0.9800	C17—H17A	0.9900
C23—H23C	0.9800	C17—H17B	0.9900
C23—C22	1.520 (4)	C1S—H1SA	0.9900
C11—H11A	0.9900	C1S—H1SB	0.9900
			
C19—Ir1—P1	94.07 (7)	C25—C26—Ir1	70.68 (15)
C19—Ir1—C25	162.50 (10)	C25—C26—H26	112.2
C19—Ir1—C26	156.41 (10)	C25—C26—C27	127.4 (2)
C19—Ir1—C30	89.60 (10)	C27—C26—Ir1	115.31 (17)
C19—Ir1—C29	83.82 (10)	C27—C26—H26	112.2
C25—Ir1—P1	90.64 (7)	C25—C32—H32A	109.0
C25—Ir1—C26	37.45 (10)	C25—C32—H32B	109.0
C25—Ir1—C30	80.07 (10)	C25—C32—C31	112.7 (2)
C25—Ir1—C29	95.89 (10)	H32A—C32—H32B	107.8
C26—Ir1—P1	98.76 (6)	C31—C32—H32A	109.0
C26—Ir1—C30	85.73 (9)	C31—C32—H32B	109.0
C26—Ir1—C29	78.59 (9)	H4A—C4—H4B	108.1
C30—Ir1—P1	158.38 (7)	C5—C4—H4A	109.5
C30—Ir1—C29	36.92 (10)	C5—C4—H4B	109.5
C29—Ir1—P1	164.70 (7)	C3—C4—H4A	109.5
C1—P1—Ir1	114.85 (8)	C3—C4—H4B	109.5
C1—P1—C13	104.19 (11)	C3—C4—C5	110.6 (2)
C7—P1—Ir1	114.70 (8)	C13—C14—H14A	109.4
C7—P1—C1	102.49 (11)	C13—C14—H14B	109.4
C7—P1—C13	108.17 (12)	H14A—C14—H14B	108.0
C13—P1—Ir1	111.52 (8)	C15—C14—C13	111.3 (2)
C19—N1—N2	113.9 (2)	C15—C14—H14A	109.4
C19—N1—C21	126.8 (2)	C15—C14—H14B	109.4
N2—N1—C21	119.3 (2)	Ir1—C30—H30	113.6
C19—N3—C20	108.6 (2)	C29—C30—Ir1	71.89 (15)
C19—N3—C22	125.4 (2)	C29—C30—H30	113.6
C20—N3—C22	125.9 (2)	C29—C30—C31	124.3 (3)
N1—C19—Ir1	129.73 (19)	C31—C30—Ir1	112.85 (17)
N1—C19—N3	102.8 (2)	C31—C30—H30	113.6
N3—C19—Ir1	126.77 (19)	C11—C10—C9	110.7 (2)
C20—N2—N1	103.0 (2)	C11—C10—H10A	109.5
P1—C1—H1	106.2	C11—C10—H10B	109.5
C6—C1—P1	115.09 (17)	C9—C10—H10A	109.5
C6—C1—H1	106.2	C9—C10—H10B	109.5
C2—C1—P1	109.51 (17)	H10A—C10—H10B	108.1
C2—C1—H1	106.2	H16A—C16—H16B	108.0
C2—C1—C6	112.9 (2)	C15—C16—H16A	109.4
P1—C7—H7	105.1	C15—C16—H16B	109.4
C12—C7—P1	114.79 (17)	C17—C16—H16A	109.4
C12—C7—H7	105.1	C17—C16—H16B	109.4
C8—C7—P1	114.64 (17)	C17—C16—C15	111.2 (2)
C8—C7—H7	105.1	N3—C22—C23	110.1 (2)
C8—C7—C12	111.0 (2)	N3—C22—H22	108.1
P1—C13—H13	105.1	N3—C22—C24	110.4 (2)
C18—C13—P1	118.41 (18)	C23—C22—H22	108.1
C18—C13—H13	105.1	C23—C22—C24	111.9 (2)
C18—C13—C14	110.1 (2)	C24—C22—H22	108.1
C14—C13—P1	111.79 (17)	C14—C15—H15A	109.5
C14—C13—H13	105.1	C14—C15—H15B	109.5
C1—C6—H6A	109.4	C16—C15—C14	110.5 (2)
C1—C6—H6B	109.4	C16—C15—H15A	109.5
H6A—C6—H6B	108.0	C16—C15—H15B	109.5
C5—C6—C1	111.1 (2)	H15A—C15—H15B	108.1
C5—C6—H6A	109.4	Ir1—C29—H29	114.1
C5—C6—H6B	109.4	C30—C29—Ir1	71.19 (15)
C7—C12—H12A	109.5	C30—C29—H29	114.1
C7—C12—H12B	109.5	C30—C29—C28	125.1 (3)
H12A—C12—H12B	108.1	C28—C29—Ir1	110.39 (18)
C11—C12—C7	110.5 (2)	C28—C29—H29	114.1
C11—C12—H12A	109.5	C6—C5—C4	112.7 (2)
C11—C12—H12B	109.5	C6—C5—H5A	109.0
C1—C2—H2A	109.2	C6—C5—H5B	109.0
C1—C2—H2B	109.2	C4—C5—H5A	109.0
H2A—C2—H2B	107.9	C4—C5—H5B	109.0
C3—C2—C1	112.1 (2)	H5A—C5—H5B	107.8
C3—C2—H2A	109.2	C32—C31—H31A	109.3
C3—C2—H2B	109.2	C32—C31—H31B	109.3
N1—C21—H21A	109.5	C30—C31—C32	111.6 (2)
N1—C21—H21B	109.5	C30—C31—H31A	109.3
N1—C21—H21C	109.5	C30—C31—H31B	109.3
H21A—C21—H21B	109.5	H31A—C31—H31B	108.0
H21A—C21—H21C	109.5	C2—C3—H3A	109.3
H21B—C21—H21C	109.5	C2—C3—H3B	109.3
Ir1—C25—H25	114.5	C4—C3—C2	111.4 (2)
C26—C25—Ir1	71.87 (15)	C4—C3—H3A	109.3
C26—C25—H25	114.5	C4—C3—H3B	109.3
C26—C25—C32	124.7 (2)	H3A—C3—H3B	108.0
C32—C25—Ir1	108.68 (18)	C22—C24—H24A	109.5
C32—C25—H25	114.5	C22—C24—H24B	109.5
C7—C8—H8A	109.5	C22—C24—H24C	109.5
C7—C8—H8B	109.5	H24A—C24—H24B	109.5
H8A—C8—H8B	108.1	H24A—C24—H24C	109.5
C9—C8—C7	110.7 (2)	H24B—C24—H24C	109.5
C9—C8—H8A	109.5	C29—C28—H28A	108.9
C9—C8—H8B	109.5	C29—C28—H28B	108.9
H23A—C23—H23B	109.5	C29—C28—C27	113.2 (2)
H23A—C23—H23C	109.5	H28A—C28—H28B	107.8
H23B—C23—H23C	109.5	C27—C28—H28A	108.9
C22—C23—H23A	109.5	C27—C28—H28B	108.9
C22—C23—H23B	109.5	C26—C27—C28	112.0 (2)
C22—C23—H23C	109.5	C26—C27—H27A	109.2
C12—C11—H11A	109.2	C26—C27—H27B	109.2
C12—C11—H11B	109.2	C28—C27—H27A	109.2
H11A—C11—H11B	107.9	C28—C27—H27B	109.2
C10—C11—C12	112.0 (2)	H27A—C27—H27B	107.9
C10—C11—H11A	109.2	C18—C17—H17A	109.2
C10—C11—H11B	109.2	C18—C17—H17B	109.2
C8—C9—H9A	109.4	C16—C17—C18	112.2 (2)
C8—C9—H9B	109.4	C16—C17—H17A	109.2
C8—C9—C10	111.0 (2)	C16—C17—H17B	109.2
H9A—C9—H9B	108.0	H17A—C17—H17B	107.9
C10—C9—H9A	109.4	F2—B1—F4	108.1 (3)
C10—C9—H9B	109.4	F3—B1—F4	109.2 (3)
C13—C18—H18A	109.5	F3—B1—F2	110.3 (4)
C13—C18—H18B	109.5	F1—B1—F4	109.7 (3)
C13—C18—C17	110.8 (2)	F1—B1—F2	109.8 (3)
H18A—C18—H18B	108.1	F1—B1—F3	109.7 (3)
C17—C18—H18A	109.5	Cl2S—C1S—H1SA	108.9
C17—C18—H18B	109.5	Cl2S—C1S—H1SB	108.9
N3—C20—H20	124.1	Cl1S—C1S—Cl2S	113.3 (2)
N2—C20—N3	111.8 (2)	Cl1S—C1S—H1SA	108.9
N2—C20—H20	124.1	Cl1S—C1S—H1SB	108.9
Ir1—C26—H26	112.2	H1SA—C1S—H1SB	107.7
			
Ir1—P1—C1—C6	−83.03 (17)	C13—P1—C1—C2	−76.91 (19)
Ir1—P1—C1—C2	45.38 (19)	C13—P1—C7—C12	81.0 (2)
Ir1—P1—C7—C12	−44.1 (2)	C13—P1—C7—C8	−49.2 (2)
Ir1—P1—C7—C8	−174.36 (15)	C13—C18—C17—C16	55.1 (3)
Ir1—P1—C13—C18	−149.88 (17)	C13—C14—C15—C16	−57.3 (3)
Ir1—P1—C13—C14	80.59 (18)	C6—C1—C2—C3	−50.3 (3)
Ir1—C25—C26—C27	−107.6 (3)	C12—C7—C8—C9	56.0 (3)
Ir1—C25—C32—C31	41.4 (3)	C12—C11—C10—C9	−56.5 (3)
Ir1—C26—C27—C28	10.0 (3)	C2—C1—C6—C5	49.8 (3)
Ir1—C30—C29—C28	102.2 (3)	C21—N1—C19—Ir1	10.7 (4)
Ir1—C30—C31—C32	13.9 (3)	C21—N1—C19—N3	−178.9 (2)
Ir1—C29—C28—C27	36.2 (3)	C21—N1—N2—C20	179.2 (2)
P1—C1—C6—C5	176.53 (17)	C25—C26—C27—C28	94.3 (3)
P1—C1—C2—C3	−179.95 (18)	C25—C32—C31—C30	−36.9 (3)
P1—C7—C12—C11	173.23 (17)	C8—C7—C12—C11	−54.8 (3)
P1—C7—C8—C9	−171.94 (18)	C8—C9—C10—C11	57.0 (3)
P1—C13—C18—C17	174.26 (19)	C18—C13—C14—C15	57.3 (3)
P1—C13—C14—C15	−168.97 (19)	C20—N3—C19—Ir1	170.16 (19)
N1—N2—C20—N3	−0.2 (3)	C20—N3—C19—N1	−0.7 (3)
C19—N1—N2—C20	−0.2 (3)	C20—N3—C22—C23	−58.8 (3)
C19—N3—C20—N2	0.6 (3)	C20—N3—C22—C24	65.3 (3)
C19—N3—C22—C23	116.4 (3)	C26—C25—C32—C31	−39.0 (4)
C19—N3—C22—C24	−119.5 (3)	C32—C25—C26—Ir1	100.6 (3)
N2—N1—C19—Ir1	−169.89 (18)	C32—C25—C26—C27	−7.0 (4)
N2—N1—C19—N3	0.6 (3)	C14—C13—C18—C17	−55.4 (3)
C1—P1—C7—C12	−169.26 (19)	C30—C29—C28—C27	−44.6 (4)
C1—P1—C7—C8	60.5 (2)	C22—N3—C19—Ir1	−5.8 (4)
C1—P1—C13—C18	−25.4 (2)	C22—N3—C19—N1	−176.6 (2)
C1—P1—C13—C14	−154.96 (18)	C22—N3—C20—N2	176.5 (2)
C1—C6—C5—C4	−53.7 (3)	C15—C16—C17—C18	−55.1 (3)
C1—C2—C3—C4	53.8 (3)	C29—C30—C31—C32	97.0 (3)
C7—P1—C1—C6	42.0 (2)	C29—C28—C27—C26	−30.5 (3)
C7—P1—C1—C2	170.42 (17)	C5—C4—C3—C2	−56.8 (3)
C7—P1—C13—C18	83.1 (2)	C31—C30—C29—Ir1	−105.8 (2)
C7—P1—C13—C14	−46.4 (2)	C31—C30—C29—C28	−3.6 (4)
C7—C12—C11—C10	55.3 (3)	C3—C4—C5—C6	57.6 (3)
C7—C8—C9—C10	−57.1 (3)	C17—C16—C15—C14	55.7 (3)
C13—P1—C1—C6	154.69 (17)		

### Hydrogen-bond geometry (Å, º)

**Table:**

*D*—H···*A*	*D*—H	H···*A*	*D*···*A*	*D*—H···*A*
C2—H2*A*···F1	0.99	2.54	3.204 (4)	124
C23—H23*C*···F1	0.98	2.51	3.137 (4)	122
C20—H20···F4^i^	0.95	2.49	3.400 (3)	161
C30—H30···F4^ii^	1.00	2.35	3.192 (3)	141
C16—H16*B*···F2^iii^	0.99	2.54	3.404 (4)	146
C22—H22···F1	1.00	2.41	3.094 (4)	125
C31—H31*B*···F3^ii^	0.99	2.35	3.330 (4)	170
C1*S*—H1*SA*···F2	0.99	2.50	3.362 (5)	145

Symmetry codes: (i) −*x*+1/2, −*y*+3/2, −*z*+1; (ii) *x*, *y*−1, *z*; (iii) *x*, −*y*+1, *z*+1/2.
